# Advances in post-translational modifications of proteins and cancer immunotherapy

**DOI:** 10.3389/fimmu.2023.1229397

**Published:** 2023-08-22

**Authors:** Yanqing Li, Runfang Zhang, Hu Hei

**Affiliations:** Department of Thyroid and Neck, Affiliated Cancer Hospital of Zhengzhou University, Henan Cancer Hospital, Zhengzhou, China

**Keywords:** post-translational modification, tumor immunotherapy, phosphorylation, ubiquitylation, succinylation

## Abstract

Protein post-translational modification (PTM) is a regulatory mechanism for protein activity modulation, localization, expression, and interactions with other cellular molecules. It involves the addition or removal of specific chemical groups on the amino acid residues of proteins. Its common forms include phosphorylation, ubiquitylation, methylation, and acetylation. Emerging research has highlighted lactylation, succinylation, and glycosylation. PTMs are involved in vital biological processes. The occurrence and development of diseases depends on protein abundance and is regulated by various PTMs. In addition, advancements in tumor immunotherapy have revealed that protein PTM is also involved in the proliferation, activation, and metabolic reprogramming of immune cells in tumor microenvironment. These PTMs play an important role in tumor immunotherapy. In this review, we comprehensively summarize the role of several types of PTMs in tumor immunotherapy. This review could provide new insights and future research directions for tumor immunotherapy.

## 1 Introduction

Tumor immunotherapy is a novel and effective treatment that overcomes tumor immune escape by activating or reversing immune cells with failed functions, thereby inhibiting or killing tumor cells (1). According to molecular mechanisms, it includes immune checkpoint inhibitors (ICIs), acceptance and commitment therapy (ACT), and monoclonal antibody therapy (2). ICIs can block the inhibitory effect of tumor cells on immune cells. In the 1990s, immunologists James P. Alison and Tasuku Honjo discovered ICIs, which marked the new era of tumor immunotherapy (3). In 2011, ipilimumab, a cytotoxic T lymphocyte antigen-4 antibody, was first used to treat melanoma (4). ACT suppresses tumors mainly by injecting specific immune cells targeting cancer cells into patients after being expanded and cultured *in vitro* (5). Anti-CD19 chimeric antigen receptor T-cell therapy (CAR-T) for B-cell lymphoma has been approved for clinical use (6). Monoclonal antibody therapy can inhibit tumors mainly by recruiting T cells to the tumor site and directly targeting tumor cells (7). Monoclonal antibodies are widely used in the field of tumor immunotherapy. Currently, the Food and Drug Administration has approved more than 100 monoclonal antibody products to enter the market (8). Immunotherapy can treat various solid and hematological tumors. New immunotherapy targets and corresponding immunotherapeutic drugs have been continuously discovered. Thus, treatment strategies for tumors have gradually shifted from inhibiting malignant proliferation and invasion of tumor cells to exploring the complex relationship between the tumor and the microenvironment around the tumor (9).

**Figure 1:**
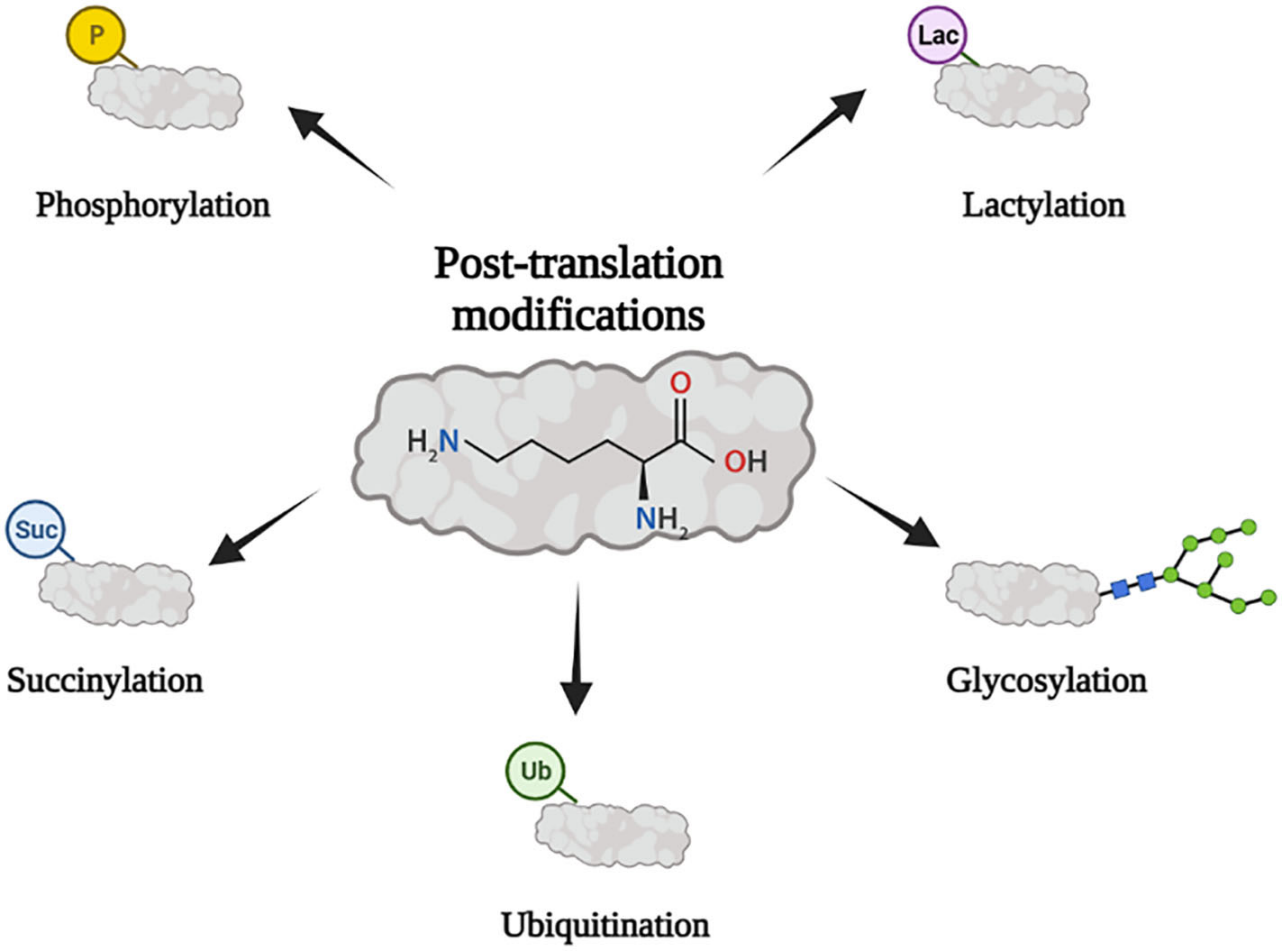
PTM is closely related to tumor immunity. Its main forms include ubiquitination, phosphorylation, glycosylation, succinylation, and lactylation.

Post-translational modification (PTM) is a covalent modification of the side chains of amino acids in translated proteins. Under physiologic and pathologic conditions, it can expand the functional diversity of proteins by regulating protein folding, activity, stability, localization, signal transduction, and binding (10). Its main forms include ubiquitin, phosphorylation, methylation, acetylation, glycosylation, and succinylation (11). It is closely related to immune cell activation, signal regulation, immune response, and tumor metabolic reprogramming (12–14). It can affect the efficacy of immunotherapy directly or indirectly by regulating immune checkpoints or remodeling tumor immune microenvironment (15–17). In this review, we summarize potential mechanisms of several types of PTMs affecting cancer development and immunotherapy (**Figure 1**).

## 2 Phosphorylation and tumor immunity

Phosphorylation is a classical and reversible PTM in which phosphate groups are covalently modified to amino acid residues after catalysis of protein kinases. It is the most common and essential PTM in eukaryotes. Approximately 30% of the proteins in mammals can be phosphorylated (18). Protein phosphorylation plays an important role in cell division, signal transduction, gene expression regulation, and protein interaction (19). Therefore, mutation of a protein phosphate site can lead to the occurrence and progression of cancer by inducing tumor cell proliferation, invasion, and metastasis and inhibiting apoptosis (18, 20). The activation or inhibition of mitogen-activated protein kinases, such as phosphoinositide 3-kinase (PI3K) and Akt kinase, and other signaling pathways is related to the phosphorylation and dephosphorylation of related proteins or enzymes in tumors, followed by regulation of the proliferation, differentiation, apoptosis, and migration of tumor cells (18).

The progression and inhibition of breast cancer are significantly related to the phosphorylation of upstream and downstream regulatory factors of nitric oxide (NO) (21). The growth and proliferation of breast cancer cells are partly induced by NO synthase, which maintains the phosphorylation of Akt and mitogen-activated protein kinase 1/2 (extracellular signal-regulated protein kinases 1 and 2 [ERK1/2]) (22, 23). However, a high NO concentration can induce apoptosis of breast cancer cells through dephosphorylation of Akt and ERK (24). Nuclear transcription factor kappa B-interacting long noncoding RNA (lncRNA) can inhibit breast cancer metastasis by blocking inhibitor of nuclear factor kappa B (NF-𝜅B) phosphorylation (25). Chao et al.’s study revealed that fructose-1,6-bisphosphatase 1 and 6‐phosphofructose‐2‐kinase/fructose‐2,6‐bisphosphatase 3 can promote breast cancer cell genesis, glycolysis, and paclitaxel resistance through phosphorylation by proviral insertion in murine lymphomas 2 (26, 27). The PI3K–Akt–mammalian target of rapamycin kinase pathway is abnormally activated in non-small cell lung cancer, and overexpression of phosphorylated Akt leads to tumor cell proliferation (28). Phosphorylation of S308 and S30 of cyclase-associated protein 1 can stimulate the proliferation, migration, and metastasis of lung cancer cells (29).

**Figure 2:**
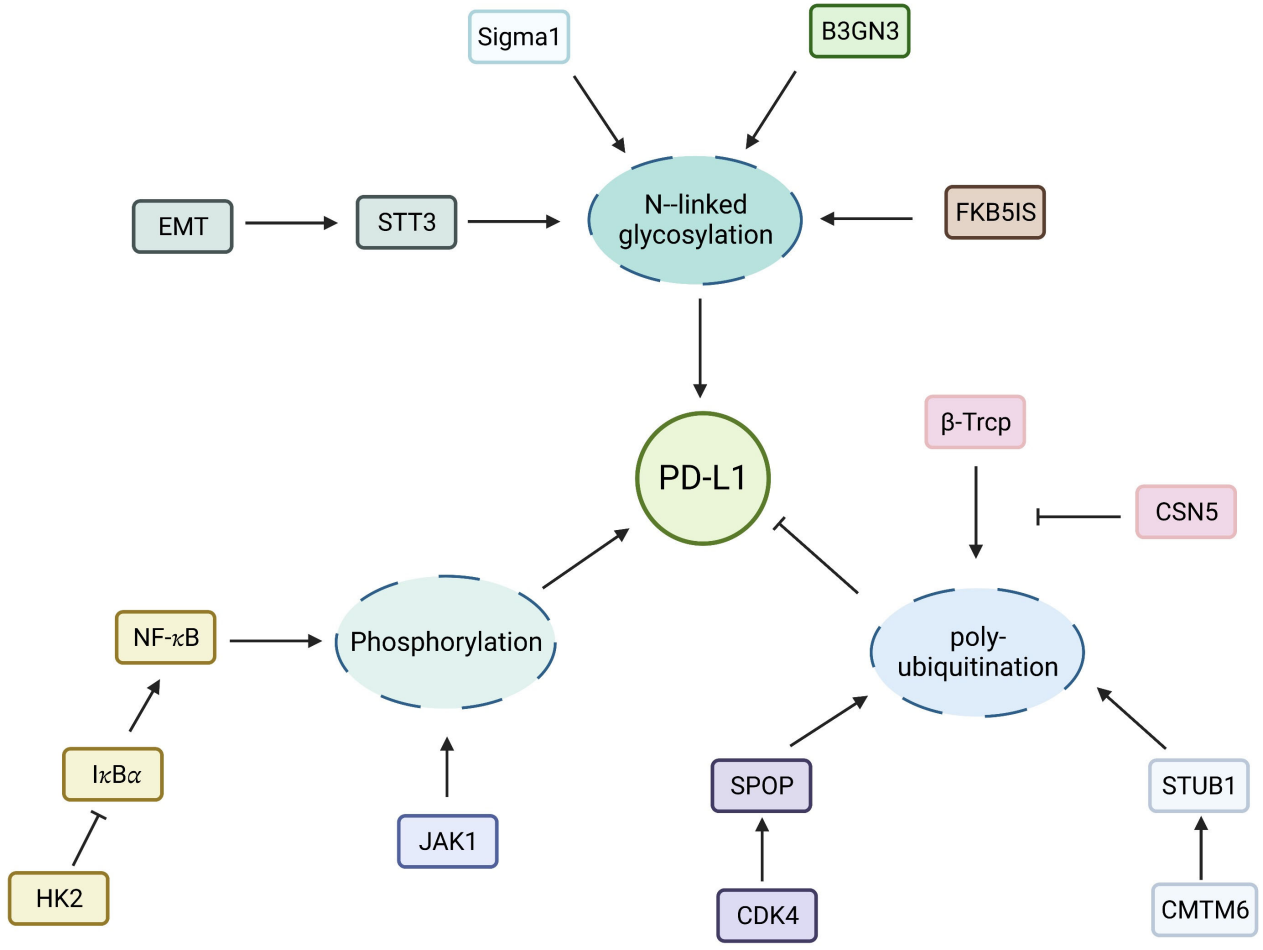
Regulation of PD-L1 by PTMs. Molecular regulation of PD-L1 N- linked glycosylation, phosphorylation and ubiquitination.

The search for immune checkpoints and ICIs is a research direction in the field of tumor immunotherapy (30). Considering the significant effect of phosphorylation on tumor characteristics, inhibitors targeting phosphokinases or phosphorylated molecules can be used as targets for tumor therapy (31). Phosphorylated transforming growth factor beta (TGF-β)-induced factor homeobox 2 (TGIF2) can induce epithelial–mesenchymal transition (EMT) and metastasis of lung adenocarcinoma, and p-TGIF2 is a potential therapeutic target for lung adenocarcinoma metastasis (32). The expression level of the CD274 molecule programmed cell death ligand-1 (PD-L1) in tumors is regulated in many aspects of translation and post-translation. Guo proved that hexokinase 2 can be used as a protein kinase to phosphorylate the Thr291 site of I𝜅Bα, thereby promoting combined protease u-calpain and I𝜅Bα and degrading I𝜅Bα, in turn promoting the entry of the NF-𝜅B transcription subunit into the nucleus and the PD-L1 expression, ultimately leading to the immune escape of the tumor (33). Combined hexokinase 2 inhibitor and PD-1 antibody in glioma can significantly improve the therapeutic effect of the PD-1 antibody. Li showed that epidermal growth factor receptor (EGFR) overexpression in tumors inhibited the phosphorylation of PD-L1 through glycogen synthase kinase-3β/α (GSK3β/α), which hindered the ubiquitination and improved the stability of PD-L1 (34). In contrast, the EGFR inhibitor osimertinib can interfere with the aforementioned process, induce ubiquitin, degrade PD-L1, and enhance the antitumor immune function of T cells (35, 36). Chen et al. found that interleukin (IL)-6 can phosphorylate PD-L1 by activating Janus kinase 1 (JAK1), in turn catalyzing PD-L1 glycosylation, enhancing its stability, and promoting tumor immune escape (37). In an animal model, the anti-IL-6 antibody combined with anti-T-cell immunoglobulin 3 induced the synergistic T-cell killing effect (37). Drugs targeting phosphokinases can be used as the focus of tumor immunotherapy. Thus, the potential application value of PTM in tumor immunotherapy has been sufficiently proven (**Figure 2**).

Some immune drugs related to phosphorylation have been developed and applied. MYCi975, a small-molecule compound, can increase the degradation of MYC by enhancing the phosphorylation of MYC on threonine-58, mediated by proteasome. In addition, it can upregulate PD-L1 and make tumors sensitive to PD1 immunotherapy (38). Simvastatin, a potential therapeutic drug for immunotherapy in colorectal cancer, inhibits the phosphorylation of YAP, mediated by the lncRNA SNHG29, and promotes antitumor immunity by inhibiting the PD-L1 expression (39). Ursodeoxycholic acid, a clinically approved compound, can enhance antitumor immunity by phosphorylating TGF-β at T282 along the Takeda G-protein-coupled receptor 5–cyclic adenosine monophosphate–protein kinase A axis and inhibiting the differentiation and activation of T_reg_ cells in mice (40). Elesclomol was identified in the differential cytotoxicity screening of the internal tool compound library. It promoted YAP phosphorylation and inhibited its nuclear accumulation through the reactive oxygen species/large tumor suppressor kinase 1 kinase signaling pathway (41). In addition, the PD-L1 expression and signal transducer and activator of transcription 3 phosphorylation increased after the nintedanib therapy for lung cancer. Nintedanib combined with αPD-L1 can enhance the therapeutic response of ICIs, further activating the tumor immune microenvironment and showing remarkable antitumor effects (42) (**Table 1**).

**Table 1:** PTM-related immunotherapy drugs.

PTM-related immunotherapy drugs		
Phosphorylation	drug	Molecular target
	osimertinib	EGFR
	MYCi975	MYC
	Simvastatin	YAP
	nintedanib	STAT3
	Metformin	PD-L1
	2DG	HK2
Ubiquitination	M435-1279	UBE2T
	ES-072	EGFR
	HUWE1	TMUB1
	Albendazole	UBQLN4
	gefitinib	EGF
	61	OTUB1/USP8
	BC-1471	STAMBP
	HOIPIN-8	LUBAC
glycosylation	NGI-1	B7-H4
	gPD-L1	PD-L1
	Stattic	PD-L2
	all-trans retinoic	MGAT3
	polyphenol resveratrol	PD-L1
	tunicamycin	PTX3
	rituximab	Fc
	SAR566658	huDS6
	gefitinib	PD-L1
	swainsonine	MAN2A1

Targeted phosphorylation performs therapeutic and predictive functions in tumor immunity. The immunohistochemistry of ERK1/2 phosphorylation can predict the overall survival rate of patients with independent recurrent glioblastoma after blocking PD-1 (43). Therefore, the development of related kits would help adjust the treatment plan of patients with PD-1 blockade.

## 3 Ubiquitylation and tumor immunity

In ubiquitination, ubiquitin molecules are covalently attached to specific residues of substrate proteins. Ubiquitination, a dynamic and reversible process, plays important roles in protein localization, metabolism, function, regulation, and degradation. Signal transduction proteins are regulated by PTM, and their ubiquitin level is second only to phosphorylation (44). Some ubiquitin-modified proteins change their function and location (45), while most are degraded by the ubiquitin–proteasome system (UPS) or lysosome degradation pathways, thus regulating various life activities, such as cell cycle, proliferation, apoptosis, differentiation, gene expression, transcriptional regulation, signal transduction, damage repair, inflammation, and immunity (46, 47). Currently, three ubiquitin enzymes are involved in ubiquitination modification: ubiquitin-activating enzyme (E1), ubiquitin-binding enzyme (E2), and ubiquitin ligase (E3). Ubiquitination is terminated by deubiquitinating proteins (DUBs). Among them, the E3 ubiquitin ligase can recognize the type of substrate protein; therefore, the specificity of the ubiquitin ligase is mainly realized by the ubiquitin ligase. The E3 ubiquitin ligase is believed to play an important role in tumor immunity (48, 49).

Casitas B-lineage lymphoma proto-oncogene b (CBL-b), as a E3 ubiquitin ligase, is an immune tolerance factor directly related to T-cell activation (50). Naramura’s study revealed that c-cbl-knockout T cells were more responsive to CD3 stimulation and promoted T-cell receptor beta variable 20/OR9-2 clearance on the cell surface, thereby inhibiting T-cell activation (51). Mutations in the ubiquitin-mediated protein degradation system can be involved in at least 10% of tumorigenesis and development (47, 52). The ubiquitin protein ligase E3 component n-recognin 5 (UBR5), an E3 ubiquitin ligase, is essential for the embryonic development of mammals (53). Elevated UBR5 expression is closely related to the survival and poor prognosis of patients with ovarian cancer (54). The E3 ubiquitin ligase regulates the activity and function of immune cells and plays an important role in regulating tumor cells and microenvironment (55).

**Figure 3:**
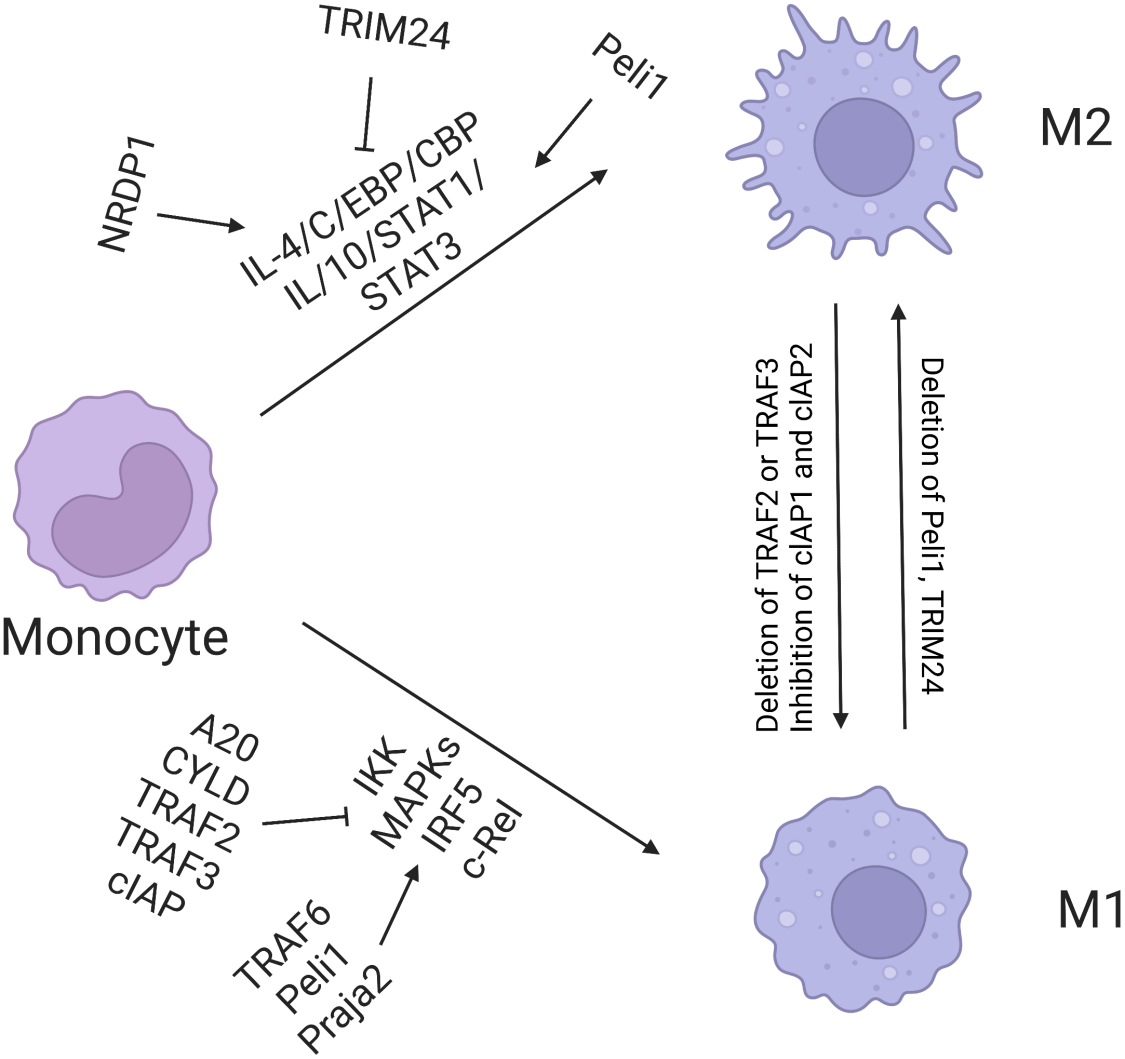
Regulation of macrophage polarization by ubiquitination. E3 ligases TRAF6, Peli1, Praja2, TRAF2, TRAF3, and cIAP promote M1 polarization, among which A20 and CYLD inhibit this process. Nrdp1 promotes the expression of M2 gene induced by IL-4 by mediating ubiquitination of K63 and activation of transcription factor C/EBP. TRIM24 inhibits M2 polarization by ubiquitination of acetyltransferase CBP.

UPS is the intracellular system responsible for protein degradation. Abnormal activation of the system accelerates the degradation of intracellular proteins. UPS can affect the survival of tumor cells by promoting the degradation of tumor-suppressor proteins, such as the tumor protein p53, or blocking the degradation of carcinogenic proteins. Song’s study proved that tumor-derived UBR5 plays a dual role in promoting tumorigenesis and affecting immune microenvironment. UBR5 can regulate tumor spheroid formation of ovarian cancer through the p53–β–catenin pathway and then enhance immunosuppression by recruiting tumor-associated macrophages (TAMs) (54). Considering the aforementioned mechanism, targeted UBR5 significantly inhibits tumor growth, eliminates the ability of ovarian cancer to resist conventional chemotherapy and immunotherapy, and significantly improves the effect of the standard treatment of ovarian cancer. In lung cancer, ubiquitin ligase interleukin 17 receptor B (CRL4) and WD repeat domain 4 promotes the progression of lung cancer through ubiquitin degradation of the promyelocytic leukemia protein. Therefore, targeted regulation of the E3 ubiquitin ligase or its substrate protein can provide new opportunities for tumor immunotherapy (56). Yu et al. showed that the ubiquitin-binding enzyme E2T (UBE2T) can promote the entry of β-catenin into the nucleus through ubiquitin degradation of the receptor for activated C kinase 1, thus promoting the occurrence and development of gastric cancer (57). The team further targeted the upstream ubiquitin-binding enzyme UBE2T to develop a small molecular inhibitor M435-1279 with low cytotoxicity that can inhibit the progression of gastric cancer *in vivo* and *in vitro* (**Figure 3**).

Owing to its role in tumor immunity, ubiquitination would be helpful in clinical immunotherapy to find new E3s and DUBs for antitumor immunomodulation and clarify their functional mechanisms. Finding and developing specific inhibitors targeting the E3 ligase and DUBs are important for clinical applications of ubiquitination. In addition to developing inhibitors, adoptive cell therapy has a clinical application potential, such as knocking-out specific E3 and DUB to improve the therapeutic effect. This method is particularly attractive for adoptive T-cell and natural killer cell therapy based on the chimeric antigen receptor.

## 4 Succinylation and tumor immunity

Succinylation modification is a type of PTM that mainly occurs in lysine residues. Compared with methylation and acetylation modification, succinylation modification has a greater effect on the structure and function of proteins. Enzymes in cell metabolism, particularly mitochondrial metabolism, are widely regulated by succinylation modification. Currently, the regulatory enzyme system of succinylation (including transferase and de-modifier enzyme) and biological function have become hot research topics.

Lu et al. first discovered histone-succinylated transferase-lysine acetyltransferase 2A, which can use succinyl coenzyme A as a substrate to catalyze the succinylation of the histone H3K79 site, thus promoting the transcription of oncogenes, tumorigenesis, and cancer development (58). In addition, glutaminase was overexpressed in pancreatic ductal carcinoma. Compared with normal cells, the growth and survival of pancreatic ductal cancer cells depended more on glutamine metabolism. In addition, glutaminase was overexpressed in pancreatic ductal carcinoma. Compared with normal cells, the growth and survival of pancreatic ductal cancer cells depended more on glutamine metabolism. Succinylation modification occurred on the glutaminase protein. Succinylation at the K311 site of the glutaminase protein was directly mediated by succinyl coenzyme A, which promoted the conversion of glutaminase from the monomer to the tetramer form. As a result, catabolism of glutamine was enhanced (59).

Tumor immunotherapy is closely related to protein succinylation modification. Tumor immune metabolism in immune cell proliferation, differentiation, response, and outcome is a research frontier worldwide. Advancements in this research would aid our understanding of immune cell biology in theory and exhibit an application prospect in maintaining immune homeostasis and tumor immunotherapy. When activated by lipopolysaccharide, macrophages consume abundant glucose, enhance glycolysis, express M1 molecular markers, and produce abundant inflammatory factor IL-1β. The mechanism involves the accumulation of succinate, the intermediate product of glucose metabolism. Succinate promotes hypoxia inducible factor-1 (HIF-1α) to enhance the transcription of IL-1β, in which the activation of pyruvate kinase M2 (PKM2), a key enzyme in glycolysis, plays an important role. Yang et al. further discovered that PKM2 is desuccinylated by sirtuin-5 (60). In SIRT5-deficient cells, the succinylation level of PKM2 increased; PKM2 transformed from the tetramer to the dimer form; and pyruvate kinase activity decreased. Dimerized PKM2 enters the nucleus and cooperates with HIF-1α to bind to the promoter region of IL-1β, which significantly enhances the transcription of IL-1β and glycolysis of macrophages. These results suggest that SIRT5 regulates macrophage metabolism and plays an important role in the malignant transformation of colitis and even colitis cancer. Metabolic changes in tumor microenvironment significantly regulate tumor immune sensitivity, but the underlying mechanism remains unclear. Cheng et al. found that tumors deficient in fumarate hydratase (FH) showed functional CD8^+^ T-cell activation, expansion, and inhibition and enhanced malignant proliferation (61). Regarding the mechanism, FH depletion in tumor cells can accumulate fumarate in tumor interstitial fluid. Elevated fumarate levels can directly succinate ZAP70 at C96 and C102 sites and eliminate the activity of infiltrating CD8^+^ T cells, thus inhibiting the activation of CD8^+^ T cells and antitumor immune response *in vitro* and *in vivo*. In addition, removing fumarate by increasing the FH expression can significantly enhance the antitumor effect of anti-CD19 CAR T cells. Thus, these findings prove the role of fumarate in controlling TCR signal transduction and suggest that the accumulation of fumarate in tumor microenvironment is a metabolic disorder of the antitumor function of CD8^+^ T cells. Potentially, fumarate depletion may be an important strategy for tumor immunotherapy.

Immune-targeted drugs for succinylation have not been confirmed in the clinical treatment of cancer. They remain in the research stage *in vivo* and *in vitro*. Nevertheless, in a previous study, 90Y-labeled succinylated streptavidin significantly inhibited the growth of breast cancer in the pre-targeted radiotherapy group (*p* < 0.05) (62). In ovarian cancer cells, inhibition of dihydrothionyl succinyltransferase, a subunit of α-KGDC in the tricarboxylic acid cycle, reduced oxidative phosphorylation and the expression and function of immunosuppressant markers in myeloid cells (63). Lactb is a positive regulator of the NF-κB signal in dendritic cells, and succinylation of the lysine 288 residue is inhibited by Suclg2. Therefore, the development of succinylated immune-targeting drugs may be a research direction for immunotherapy.

## 5 Lactylation and tumor immunity

Lactic acid is a metabolite of cellular glycolysis. However, it has been considered a simple cellular energy substance and metabolite. Its regulatory role in biological function has been unknown. Lactic acid-mediated protein PTM lactylation plays a regulatory role in immune cells and cancer metabolism.

Regarding the tumor metabolism, Zhao et al. found that lactic acid accumulated during metabolism can be used as a precursor to induce lactylation of histone lysine and participate in the homeostasis regulation of M1 macrophages infected by bacteria (64). In addition to a study on the epigenetic regulation of histone lactic acid modification, Gao et al. drew a panoramic map of lactic acid modification in hepatocellular carcinoma for the first time. Lactylation occurs in histones and plays a global regulatory role in hepatocellular carcinoma by affecting widely distributed non-histone proteins. E1A-binding protein p300. Histone deacetylase causes the activation and deactivation of non-histone lactylation (65). Lu et al. found that the metabolite lactic acid affects the tumor microenvironment and promotes tumorigenesis by regulating the lactylation of M protein Lys72 in T_reg_ cells and enhancing TGF-β signal transduction, which provides a new theoretical basis for cancer immunotherapy by targeting T_reg_ cells (66). Zhao et al. found that activated macrophages play an important role in ulcerative colitis. Lactic acid can enhance histone H3K18 lactylation in macrophages, inhibit macrophage coking, and restore intestinal immune function (67). In addition, Zhang et al. found that lactic acid-mediated lactylation of PKM2 at the K62 site can enhance the pyruvate kinase activity of PKM2 to inhibit the Warburg effect and ultimately promote the transition of macrophages from the proinflammatory to the repair phenotype (68). The underexpression of sirtuin 3 in hepatocellular carcinoma promotes the lactylation of cyclin E2, which in turn promotes tumor progression, and sirtuin 3 is a potential therapeutic target for hepatocellular carcinoma (69).

The lactate score model can be used to predict tumor immune escape (70). A lactic acid-related model study on gastric cancer revealed numerous infiltrated immune cells (macrophages to the highest degree), characterized by an increased lactic acid score. ICIs showed a decreased response rate in gastric cancer with a high lactate score. Tumors with a high lactate fraction have high tumor immune dysfunction, implying higher risks of immune escape and dysfunction. These findings indicate that the lactate score can be used to predict malignant progression and immune evasion of gastric cancer. However, the application of lactic acid drugs in clinical tumor immunity remains under development.

## 6 Glycosylation and tumor immunity

Glycosylation is an important protein PTM in which O-linked N-acetylglucosamine (O-GlcNAcylation) refers to addition of monosaccharide modification to serine and/or threonine residues of protein in cells, which is the most common glycosylation form in eukaryotes (71). This modification is a highly dynamic modification method, which would change with the nutritional status in cells and extracellular stimuli. It widely occurs in intracellular proteins and regulates important biological processes, such as gene transcription, signal transduction, protein synthesis, and metabolic reprogramming.

As early as 1991, Crowley et al. discovered the effect of non-enzymatic glycosylation on the function of mesangial cells (72). Khidekel discovered a new strategy to monitor the glycosylation kinetics of O-GlcNAc using protein omics based on quantitative mass spectrometry (73). Fogel et al. found that site-specific N-glycosylation affects the structure and function of binding synaptic cell adhesion molecule interaction (74). In addition, some studies have summarized the current knowledge of immunoglobulin glycosylation and paid special attention to the research and vaccination for infectious diseases, considered to be a field with many interesting opportunities (75). Hu et al. analyzed 83 high-grade serous ovarian cancer and 23 non-tumor tissues prospectively with comprehensive protein omics and glycochemistry. Tyagi et al. comprehensively summarized the discovery of RNA glycosylation, conceptually understood its previous potential discovery and its biological consequences, and explained the dynamic impact of this modification on its molecular versatility, determining the immunological fate of cancer and the potential impact of glycosylation on cell interaction, signal transduction, immunomodulation, cancer escape, and proliferation (76). Shi et al. found that glucose metabolism in TAM was modified by enhancing O-GlcNAcylation, promoting tumor metastasis and chemotherapy resistance. They revealed that M2-like TAM is the immune cell subgroup with the strongest glucose uptake ability in tumor microenvironment and discovered the new function of O-linked N-acetylglucosamine transferase located in lysosomes (77). They clarified the significance of competitive uptake and utilization of glucose by TAM, particularly M2-like TAM, in shaping cell-specific tumor-promoting function, which provided a potential target for tumor treatment.

The star molecule of glycosylation in tumor immunotherapy is PD-L1. Glycosylation can stabilize PD-L1, which prevents PD-L1 from degradation of 26S proteasome mediated by GSK3β, thus enhancing its interaction with PD-1 on CD8^+^ T cells. In addition, the catalytic subunit of oligo-glycosyltransferase STT3 transfers the core glycan structure to PD-L1, which leads to EMT. Combined PD-L1 and PD-1 is also influenced by the glycosylation of PD-L1. In a study on EGF/EGFR signal transduction, PD-L1/PD-1 interaction requires β 1,3-N- acetylglucosamine transferase 3 (B3GNT3) to mediate the glycosylation of poly-N-acetyllactosamine on PD-L1 N192 and N200. The 4T1 cells lacking B3GNT3 expression grew in severe combined immunodeficiency mice, but not in immunocompetent BALB/c mice (78). Therefore, glycosylation targeting PD-L1 is a breakthrough in tumor immunotherapy.

## 7 Conclusions

PTMs are chemical changes that occur after protein synthesis that play a vital role in regulating protein function, stability, localization, and interactions. In addition to the types of PTMs summarized above, other PTMs play a vital role in tumor immunity. Currently, immune-related PTM drugs mostly include phosphorylation inhibitors. In addition, some PTM models can predict tumor immune evasion, such as the lactic acid score model. Immune-targeted drugs for succinylation have not been confirmed in the clinical treatment of cancer and remain in the research stage *in vivo* and *in vitro*. In addition to developing inhibitors, adoptive cell therapy carries clinical application potential, such as knocking-out specific PTM-related proteins to improve the therapeutic effect. This method is particularly attractive for adoptive T-cell and natural killer cell therapy based on the chimeric antigen receptor. This may be the direction of PTM immunotherapy in the future. In general, protein PTM is a regulator of tumor immunity. Its disorders affect various immune processes, including T-cell activation, immune checkpoint regulation, cytokine production, and immune cell interaction in the tumor microenvironment. Understanding its role in tumor immunity may provide insights for the development of new immunotherapies and targeted therapies for cancer.

## Author contributions

Conceptualization: YL and HH. Formal analysis: YL. Investigation: HH. Resources: YL. Data curation: HH. Writing—original draft preparation: YL. Writing—review and editing: HH. Visualization: HH. Supervision: RZ. Project administration: RZ. Funding acquisition: YL. All authors have read and agreed to the published version of the manuscript.
